# Dispensers and the Insurance Bill

**Published:** 1911-11-18

**Authors:** 


### The Editor's Letter Box.

### DISPENSERS AND THE INSURANCE BILL.

"Patiens" writes : Your correspondent "An Apothecary's Assistant," in his letter which appears in your issue of the 4th inst., whilst stating the case generally for the apothecary's assistant in a remarkably forceful and lucid manner, makes a statement which will cause, I am sure, a great feeling of resentment in the minds of all who know and appreciate the untiring efforts on behalf of the certificated dispenser of our worthy secretary.

To say that the Association of Certificated Dispensers and its secretary have been asleep during this critical period is to confess to a complete, and, under the circumstances, reprehensible, ignorance of the work of both. Allow me to assure your correspondent that the absolute converse is the case. In matters of this kind he should know that the best results are not attained by impolitically blazoning our work to the four winds of heaven, but by persistent and intelligent methods, and if your correspondent, who I shrewdly suspect does not live a great many miles from Suffolk, would take the trouble to inquire from the proper quarter, I am sure he would be satisfied that judgment has by no manner of means gone by default, but rather that he would find activities in full action, at no time more so than at present, of which he has taken no count.

